# Gene Expression Profiling of the Local Cecal Response of Genetic Chicken Lines That Differ in Their Susceptibility to *Campylobacter jejuni* Colonization

**DOI:** 10.1371/journal.pone.0011827

**Published:** 2010-07-28

**Authors:** Xianyao Li, Christina L. Swaggerty, Michael H. Kogut, Hsin-I Chiang, Ying Wang, Kenneth J. Genovese, Haiqi He, Huaijun Zhou

**Affiliations:** 1 Department of Poultry Science, Texas A&M University, College Station, Texas, United States of America; 2 Southern Plains Agricultural Research Center, United States Department of Agriculture, Agricultural Research Service, College Station, Texas, United States of America; Texas A&M University, United States of America

## Abstract

*Campylobacter jejuni* (*C. jejuni*) is one of the most common causes of human bacterial enteritis worldwide primarily due to contaminated poultry products. Previously, we found a significant difference in *C. jejuni* colonization in the ceca between two genetically distinct broiler lines (Line A (resistant) has less colony than line B (susceptible) on day 7 post inoculation). We hypothesize that different mechanisms between these two genetic lines may affect their ability to resist *C. jejuni* colonization in chickens. The molecular mechanisms of the local host response to *C. jejuni* colonization in chickens have not been well understood. In the present study, to profile the cecal gene expression in the response to *C. jejuni* colonization and to compare differences between two lines at the molecular level, RNA of ceca from two genetic lines of chickens (A and B) were applied to a chicken whole genome microarray for a pair-comparison between inoculated (I) and non-inoculated (N) chickens **within** each line and **between** lines. Our results demonstrated that metabolism process and insulin receptor signaling pathways are key contributors to the different response to *C. jejuni* colonization between lines A and B. With *C. jejuni* inoculation, lymphocyte activation and lymphoid organ development functions are important for line A host defenses, while cell differentiation, communication and signaling pathways are important for line B. Interestingly, circadian rhythm appears play a critical role in host response of the more resistant A line to *C. jejuni* colonization. A dramatic differential host response was observed between these two lines of chickens. The more susceptible line B chickens responded to *C. jejuni* inoculation with a dramatic up-regulation in lipid, glucose, and amino acid metabolism, which is undoubtedly for use in the response to the colonization with little or no change in immune host defenses. However, in more resistant line A birds the host defense responses were characterized by an up-regulation lymphocyte activation, probably by regulatory T cells and an increased expression of the NLR recognition receptor NALP1. To our knowledge, this is the first time each of these responses has been observed in the avian response to an intestinal bacterial pathogen.

## Introduction

*Campylobacter jejuni* (*C. jejuni*) is one of the main food-borne bacterial pathogens of humans in developed countries [1]. Chickens are a major reservoir of *C. jejuni* with contaminated under-cooked or raw chicken as one of the main sources of human *Campylobacter* infections [1]. In the U.S., the cost of campylobacteriosis is estimated to be $1.5–8.0 billion annually. Reducing *Campylobacter* contamination in food could save up to $5.6 billion annually [2], [3].

The ability of *C. jejuni* to colonize in chickens has been well documented and the cecum is the primary site of colonization [4]. Studies show that the host genetic background plays an important role in the response to *C. jejuni* infection [5], [6], [7]. We have previously shown different numbers of *C. jejuni* in cecal content at day 7 post-inoculation (pi) between two broiler lines (lines A vs. line B) where line A is more resistant to cecal colonization by *C. jejuni* compared to line B [7]. Understanding molecular mechanisms contributing to resistance to *C. jejuni* colonization will be essential for the improvement of genetic resistance to *C. jejuni* colonization in the chicken. Therefore, cecum including cecal tonsil (one of major lymphoid tissues interacting with *C. jejuni*) at day 7 pi were collected to elucidate underlining mechanisms affecting resistance and the local host response to *C. jejuni* colonization.

Gene expression changes following *C. jejuni* inoculation has focused on cytokines and chemokines in human and chicken by quantitative real-time PCR [8], [9], [10], [11]. High-throughput microarray technology can provide a comprehensive view of global gene expression changes in the host during a *C. jejuni* inoculation at a given point in time under uniform experimental condition [12] as was previously carried out in the response to *Salmonella* infection in chickens [13], [14], [15], [16]. The available chicken genomic sequence [17] provides an opportunity to study the large-scale gene expression profiling of chickens in the response to *C. jejuni* inoculation. We report here the use of a chicken-specific 44K Agilent microarray [18] to profile host gene expression transcription of ceca between two lines of chickens and characterize their host response following *C. jejuni* inoculation.

Therefore, the objectives of the present study were twofold: (1) to evaluate the differences in gene expression **between** these two lines of chickens that differ in their resistance to *C. jejuni* cecal colonization, and (2) to identify differentially expressed genes **within** lines following *C. jejuni* inoculation when compared to the non-inoculated controls.

## Results

### 1. Identification of differentially expressed genes *between* lines

**Figure 1:**
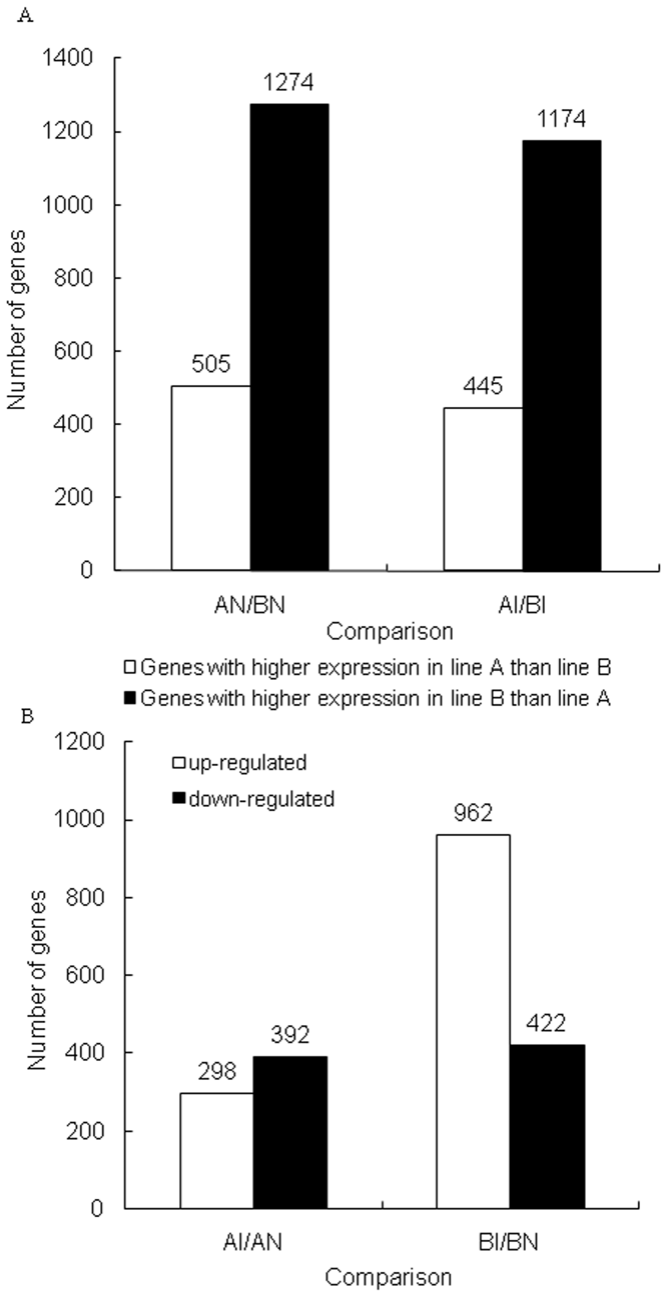
Number of significantly differentially expressed genes between comparisons. A: Number of genes with higher expression in one line (A or B) than the other line (B or A) when comparing between lines A and B. White bar represents number of genes with higher expression in line A than line B; Black bar represents number of genes with higher expression in line B than line A. B: Number of up and down-regulated genes following *C. jejuni* inoculation within each line (A or B) when comparing inoculated with non-inoculated control chickens. White bar represents number of up-regulated genes; Black bar represents number of down-regulated genes.

The number of *C. jejuni* colonization in cecal content at day 7 pi in line A (1.39 log10 cfu (colony-forming unit)) is significantly lower than that in line B (3.50 log10 cfu) [7]. To compare genetic difference between these two lines, the significantly expressed genes between line A and line B in both inoculated and non-inoculated chickens were identified (Fig. 1A).

#### A. Comparison of gene profile of ceca between lines of non-inoculated birds

Initially, we compared the gene expression profile of the ceca of lines A and B of non- inoculated controls (AN vs. BN), and found 1,779 genes significantly expressed between line A and line B with a false discovery rate (FDR) of 0.577 (Fig. 1A). Of the 1,779 genes, 505 genes were more highly expressed in line A than line B and 1,274 were more highly expressed in line B than line A. Of the more highly expressed genes in line B, 774 had a fold-change >2, and 30 genes had a fold-change >10. The highest fold-change (152) was observed for the dopey family member 1 (DOPEY1, CR353647) gene. Among the genes higher expressed in line A, 368 had a fold-change >2 and 7 genes had a fold-change >10. The highest fold-change (134) was observed for the NDC80 kinetochore complex component, homolog (*S. cerevisiae*) (NUF2, AJ720907) gene (Table S1).

#### B. Comparison of gene profile of ceca between lines of C. jejuni inoculated birds

**Figure 2:**
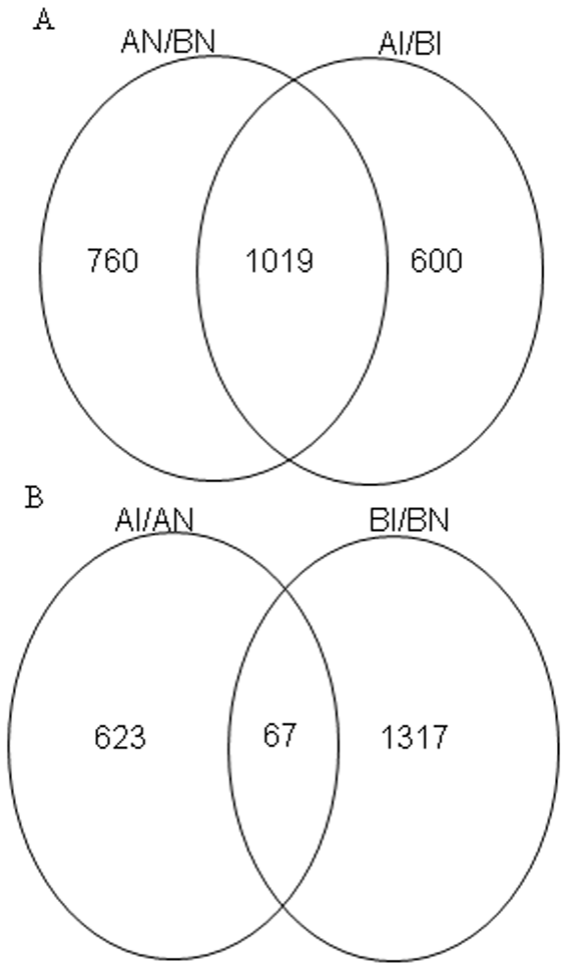
Venn diagram showing the number of differentially expressed genes overlapped in different comparisons. A: Number of genes overlapped between lines A and B of inoculated and non-inoculated birds. B: Number of genes overlapped between inoculated birds and non-inoculated within A line and B line.

Following inoculation with *C. jejuni*, we compared the changes in the gene expression profile of the ceca of lines A and B. The results showed that 1,619 genes were differentially expressed with a FDR of 0.288, and 1,174 had higher expression in line B than line A. Among those genes, 640 had a fold-change >2, and 25 genes had a fold-change >10. As observed in the AN/BN comparison, the highest fold-change (128) was the DOPEY1 gene (CR353647). Of the genes that had higher expression in line A, 311 had fold-change >2 and 7 genes had fold-change >10. The highest fold-change was found for the BX265589 (a chicken EST) (Table S2). Greater than 50% of those differentially expressed genes were shared between the AN/BN and AI/BI comparisons (Fig. 2A).

### 2. Identification of differentially expressed genes between inoculated and non-inoculated birds *within* lines

#### A. Within line A

The comparison of inoculated vs. non-inoculated chickens within line A (AI/AN) showed 690 genes significantly expressed with a FDR of 0.174. Of 690 genes, 392 were down-regulated and 298 were up-regulated (Fig. 1B). The highest fold-change (4.0) was observed for the AJ741056 (a chicken EST). Among the down-regulated genes, 46 had a fold-change >2. In the up-regulated genes, 34 had a fold-change >2 (Table S3).

#### B. Within line B

In the comparison of inoculated vs. non-inoculated chickens within line B (BI/BN), 1,384 genes were differentially expressed with a FDR of 0.182. More genes were up-regulated (962) than down-regulated (422) (Fig. 1B). Of down regulated genes, 62 had a fold-change >2 with the highest fold-change (5.34) found for the CR339022 (a chicken EST). Among up-regulated genes, 82 had a fold-change >2 with the highest fold-change (3.93) observed for the TC225367 (a chicken EST) (Table S4).

To compare differentially expressed genes of inoculated vs. non-inoculated between A line and B line, there were 67 genes shared (28 up-regulated and 36 down-regulated genes with consistent expression direction and three genes with opposite expression direction) (Fig. 2B, Table S5).

There were five genes overlapped in all four comparisons (AN/BN, AI/BI, AI/AN, BI/BN). They were SOCS3 (AF424806), IL-1β (Y15006), and K60 (Y14971), and the other two were non-annotated chicken ESTs.

### 3. Gene functional analysis

Functional category enrichment based on the gene ontology (GO) was evaluated on the differentially expressed genes between two different lines and between inoculated and non-inoculated within lines (up- and down-regulated) by Database for Annotation, Visualization and Integrated Discovery (DAVID) 2008 [19]. Three categories are included in GO: biological process (BP), molecular function (MF), and cellular component (CC). Each of these categories are assigned independently [20]. Due to significant relevance of BP, only functional clusters belonging to this category are presented in the current study.

#### A. Functional analysis of genetic difference between lines

**Figure 3:**
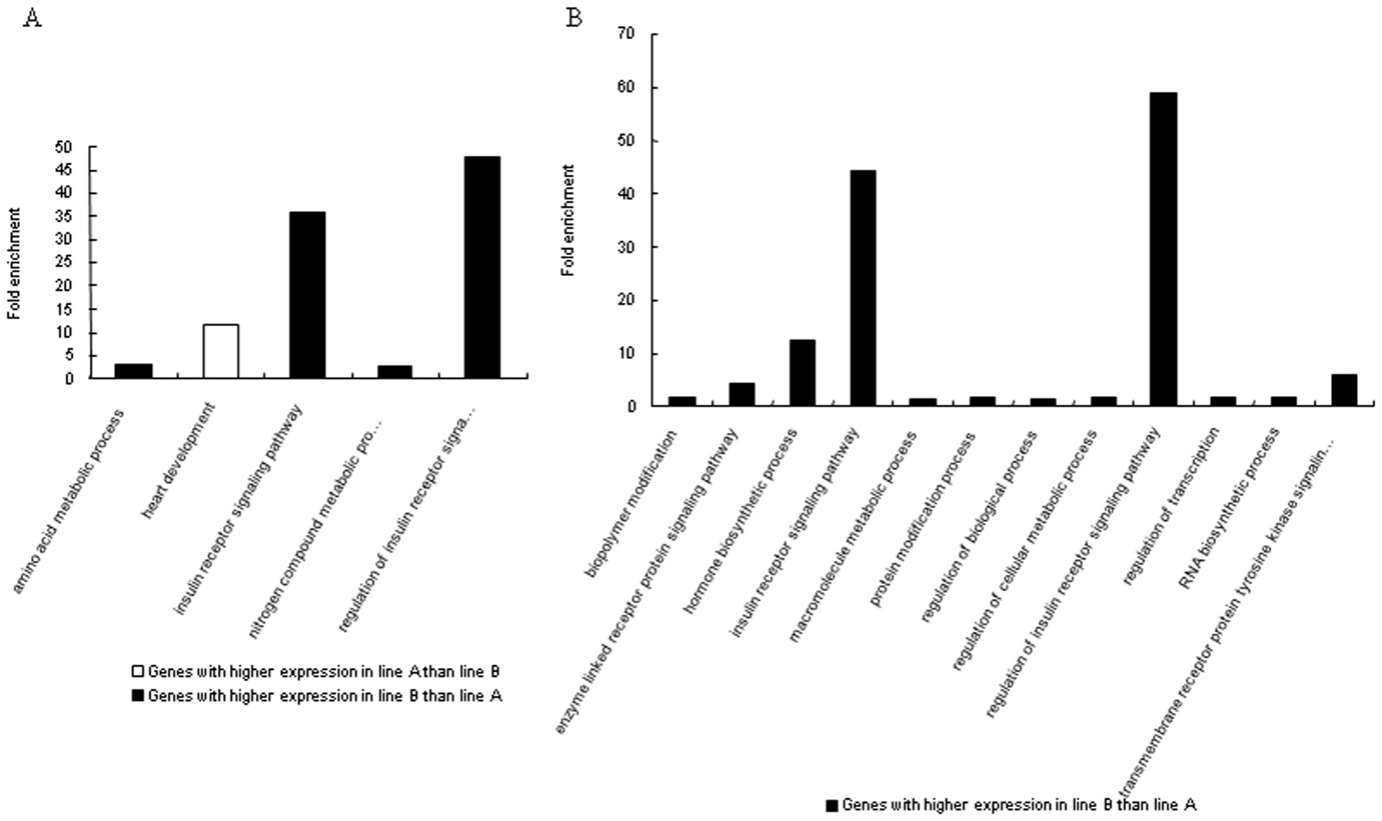
Enriched BP GO terms for significantly expressed genes *between* genetic lines. A: Enriched GO terms in the comparison of AN/BN. Note: regulation of insulin receptor signal… represents regulation of insulin receptor signaling pathway. B: Enriched GO terms in the comparison of AI/BI. Note: transmembrane receptor protein tyrosine kinase signalin…represents transmembrane receptor protein tyrosine kinase signaling pathway.

*Comparisons of non-inoculated birds between lines*: In the comparison of AN/BN, for the genes with higher expression in line B, four enriched GO terms were observed. These included amino acid metabolic process, insulin receptor signaling pathway, nitrogen compound metabolic process and regulation of insulin receptor signal signaling pathway with fold enrichments of 3.0, 35.7, 2.7, and 47.6, respectively. For the genes with higher expression in line A, one enriched GO term, heart development, was significantly enriched with fold enrichment of 11.7 (Figure 3A).

*Comparisons of inoculated birds between lines*: Although fewer differentially expressed genes were observed in the comparison of AI/BI, more enriched GO terms were obtained (Figure 3B). All significantly enriched GO terms were from genes higher expressed in line B. The enriched GO terms could be roughly grouped into two clusters. The first cluster is comprised of cellular processes and their regulation (biopolymer modification, hormone biosynthetic process, macromolecule metabolic process, protein modification process, regulation of cellular metabolic process, and RNA biosynthetic process). The second cluster centers on signaling pathways including enzyme linked receptor protein signaling pathway, insulin receptor signaling pathway, regulation of insulin receptor signaling pathway, and transmembrane receptor protein tyrosine kinase signaling pathway. Higher fold enrichment (>10) was detected in hormone biosynthetic process, insulin receptor signaling pathway and regulation of insulin receptor signal signaling pathway (12.6, 44.3, and 59.0, respectively).

#### B. Functional analysis associated with C. jejuni colonization within lines

**Figure 4:**
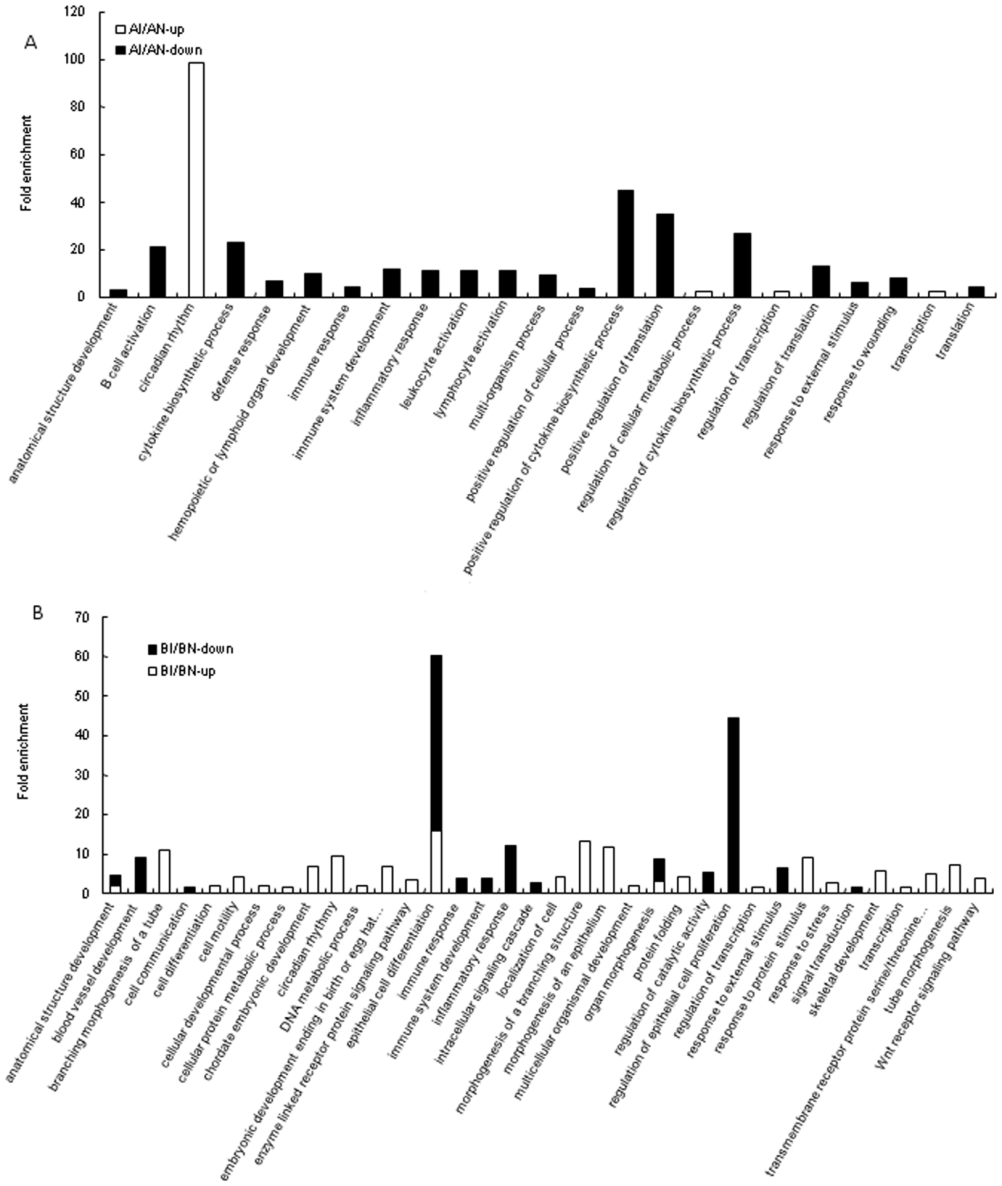
Enriched BP GO terms for significantly expressed genes between inoculated and non-inoculated birds *within* lines. A: Enriched BP GO terms in the comparison of AI/AN. B: Enriched BP GO terms in the comparison of BI/BN. Note: embryonic development ending in birth or egg hat… represents embryonic development ending in birth or egg hatch; transmembrane receptor protein serine/threonie… represents transmembrane receptor protein serine/threonine kinase signaling pathway.

For the comparison of AI/AN, more enriched GO terms were found in the down-regulated genes than up-regulated ones (Figure 4A). Nineteen enriched GO terms were from down-regulated genes. A majority of these enriched GO terms play a role in the immune system, and include B cell activation, cytokine biosynthetic process, defense response, hemopoietic or lymphoid organ development, immune response, immune system development, inflammatory response, leukocyte activation, lymphocyte activation, positive regulation of cytokine biosynthetic process, regulation of cytokine biosynthetic process, response to external stimulus, and response to wounding. The remaining enriched GO terms were comprised of development and metabolic related process such as anatomical structure development, multi-organism process, and regulation of cellular metabolic process. For the up-regulated genes, only circadian rhythm was significantly enriched.

For line B comparison (BI/BN), more enriched GO terms were found in the up-regulated genes following *C. jejuni* inoculation (Figure 4B). These functional terms could be roughly grouped into five clusters: (1) development and morphogenesis including anatomical structure development, branching morphogenesis of a tube, cell differentiation, cellular developmental process, chordate embryonic development, embryonic development ending in birth or egg hatching, epithelial cell differentiation, morphogenesis of a branching structure, morphogenesis of an epithelium, multicellular organismal development, organ morphogenesis, skeletal development, and tube morphogenesis; (2) immune response to protein stimulus and response to stress; (3) cell communication and cell motility; (4) protein and DNA metabolic process; and (5) signaling pathways such as enzyme linked receptor protein signaling pathway and Wnt receptor signaling pathway. Enriched functional terms found in the down-regulated genes included immune response, immune system development, inflammatory response, response to external stimulus, cell communication, and signal transduction.

### 4. Immune-related genes

Immune-related genes are biologically important for the host response to antigens. Based on our knowledge and information available, 426 postulated immune-related genes [21], [22], [23] and gene products were identified in the 44K chicken Agilent array used in the current study.

#### A. Significantly expressed immune-related genes between genetic lines

Due to differences in resistance to *C. jejuni* colonization between lines A and B, it is expected that some immune-related genes would be differentially expressed between the two lines. The results showed 17 immune-related genes were significantly expressed in the comparison of AN/BN with fold-change ranging from 1.57 (chemokine-like ligand 1, CF258095) to 4.41 (CHT28, X67915) (Table 1). The majority of immune-related genes had higher expression in BN than AN. Only two immune response genes (complement receptor 1, AB109024 and NALP1, XM_415289) had a significantly greater fold change in expression in AN than BN (Table 1).

**Table 1:** Fold-change of significantly differentially expressed immune-related genes *between* genetic lines in the microarray results (*P*<0.01).

Accession No.	Gene description	AN/BN	AI/BI
AB025103	Immunoglobulin J chain	−3.88	
AB109024	Complement receptor1	1.86	1.71
AF424806	Suppressor of cytokine signaling 3 (SOCS3)	−2.28	−2.31
AF498236	Suppressor of cytokine signaling 2 (SOCS2)		−2.25
AJ720544	Interleukin F2	−2.04	−1.77
AJ720982	Chemokine (C-Cmotif) receptor8	−4.33	−3.01
AJ852017	Interleukin -7 (IL7)	−3.42	−1.85
AY460177	MRAS	−2.85	
AY621314	β -defensin12		3.59
BG625680	Putative E-selectin	−1.79	−1.54
BU344261	TANK-binding kinase 1 (TBK1)		−2.78
BU376898	CD135	−2.19	−2.53
BX931297	Cytokine like protein 17		7.54
CF258095	Chemokine-like ligand 1	−1.57	
CR352545	Granulocyte-macrophage colony-stimulating factor		−1.64
CR388516	β -defensin10		3.47
XM_415289	NALP1 (LOC416998)	3.30	2.16
L18784	Transforming growth factor, beta receptor II		3.30
X67915	Lymphocyte surface marker mammalian CD28 homologue (CHT28)	−4.41	−2.80
X71786	Integrin beta 2 (ITGB2)	−2.35	
Y14971	CXC chemokine K60 (K60)	−2.02	−2.09
Y15006	Interleukin-1beta (IL1b)	−3.51	−3.10
Z22726	CD8 alpha	−3.91	−4.03
Z26484	CD8 beta	−2.11	−2.05

In the comparison of AI/BI, 20 immune-related genes were significantly expressed with the fold-changes ranging from 1.54 (putative E-selectin, BG625680) to 7.54 (cytokine like protein 17, BX931297) (Table 1). The majority of those immune-related genes (14 out of 20) had higher expression levels in BI than AI. There were 13 immune-related genes shared between AN/BN and AI/BI with the same direction of the regulation (up- or down-regulated).

#### B. Significantly expressed immune-related genes between inoculated and non-inoculated birds within lines

A list of immune-related genes responding to *C. jejuni* colonization is shown in Table 2. For the comparison of AI/AN, 17 genes were differentially down-regulated. The highest fold-change (1.92) was observed for IL8 (M16199) while one of the toll-like receptors, TLR7 (AJ720504), was differentially expressed with a fold-change of 1.27. Interleukin 3 (IL3) regulated nuclear factor (AF335427) was the only up-regulated gene with a fold-change of 1.36.

**Table 2:** Fold-change of significantly differentially expressed immune-related genes between inoculated and non-inoculated chickens *within* lines in microarray results (*P*<0.01).

Accession No.	Gene description	AI/AN	BI/BN
AB015289	B cell adaptor containing SH2 domain	−1.17	
AB109024	Complement receptor1		1.26
AF074248	Signal transducer and activator of transcription 5	−1.20	
AF335427	Nuclear factor, interleukin 3 regulated	1.36	
AF424806	Suppressor of cytokine signaling 3 (SOCS3)	−1.60	−1.58
AJ450829	Chemokine receptor 5 (CXCR5)	−1.80	
AJ719741	CKLF-like MARVEL transmembrane domain containing 7	−1.29	−1.27
AJ719814	B cell antigen receptor associated protein	−1.39	
AJ720236	NCK adaptor protein 2		1.15
AJ720504	Toll-like receptor 7 (TLR7)	−1.27	
AJ720845	RAS guanyl releasing protein 3 (calcium and DAG-regulated)		−1.35
AJ721122	Mitogen-activated protein kinase kinase 5 (MAPKK5)		1.18
AJ843261	Transporter associated with antigen processing 1 (TAP1)		1.28
AJ851659	CD80 antigen	−1.22	
AJ851740	Ankyrin repeat and SOCS box-containing 7		1.35
AY566700	Growth/differentiation factor-9		2.02
BU308587	Heat-shock protein (HSP70)		1.29
BX934914	Interleukin 22 receptor alpha		1.25
CK610423	Chemokine ah221 (CCL11)		−2.00
CR338861	CKLF-like MARVEL transmembrane domain containing 4		1.28
CR390308	Glioma Amplified Sequence 41		−1.42
CR406783	Ficolin (collagen/fibrinogendomaincontaininglectin) 2	−1.29	−1.26
CR523215	Natural killer cell receptor 2B4		−1.33
CR523828	Suppressor of cytokine signaling 5 (SOCS5)		1.18
D16367	NFkB-2; Nuclear factor NF-kBp52/p100	−1.27	
DQ267901	Toll- like receptor 15 (TLR15)		−1.43
XM_415289	NALP1 (LOC416998)		1.53
L06109	Purinergic receptor P2Y, G-protein coupled 5 (P2RY5)	−1.18	
M16199	Interleukin 8 (IL8)	−1.92	−1.95
Y12011	CD5	−1.50	
Y14971	CXC chemokine K60 (K60)	−1.75	−1.69
Y15006	Interleukin-1beta (IL1b)	−1.57	−1.78
Y18692	Chemokine K203 (K203)	−1.46	

For the comparison of BI/BN, 22 genes were differentially expressed with 11 of the immune-related genes up-regulated following *C. jejuni* inoculation including Mitogen-activated protein kinase kinase 5 (MAPKK5, AJ721122), Heat-shock protein (HSP70, BU308587), Interleukin 22 receptor alpha (BX934914), and Suppressor of cytokine signaling 5 (SOCS5, CR523528).

### 5. Validation of gene expression from microarray analysis by quantitative real-time PCR

Quantitative real-time PCR (qRT-PCR) was performed to validate the microarray data and the same RNA samples were used. Ten differentially expressed genes associated with the immune response and circadian rhythm functional terms were selected for the validation by qRT-PCR (The primer sequences were listed in Table S6). The results showed that nine of ten genes selected for validation were consistent with the results obtained from the microarray in terms of significance and direction of the regulation. The STAT5B showed up-regulation in both microarray and qRT-PCR results, but not statistical significance in qRT-PCR result. Due to the increased sensitivity of qRT-PCR compared to the microarray, the fold-change in the qRT-PCR results were higher than that observed from the microarray analysis (Table 3).

**Table 3:** Comparison of gene expression levels between microarray and qRT-PCR.

Comparison	AI/AN		BI/BN		AN/BN		AI/BI	
MethodGenes	qRT-PCR	Micro-array	qRT-PCR	Micro-array	qRT-PCR	Micro-array	qRT-PCR	Micro-array
IL-1b	−2.03*	−1.57*	−2.21*	−1.78*	−3.77*	−3.51*		
SOCS3	−1.76*	−1.60*					−2.71*	−2.31*
K60			−2.21*	−1.69*	−2.26*	−2.02*		
IL-8	−2.32*	−1.92*						
GAL10							8.51*	3.47*
CD5	−1.92*	−1.50*						
CD80	1.94*	−1.22*						
STAT5B	−1.34	−1.20*						
GHRL			3.80*	2.08*				
CLOCK	1.72*	1.29*	1.66*	1.40*				

Note: Fold-change was listed in the table.

*represents the gene significantly differentially expressed in the comparison (*P*<0.05 in RT-PCR result, *P*<0.005 in microarray).

## Discussion

In the present study, a genome-wide gene expression profile of host response to *C. jejuni* inoculation in the ceca from two genetically different broiler lines were studied using a chicken DNA microarray. Expression profiling in the comparison *between* two genetic lines and their response to *C. jejuni* inoculation *within* each line were described in the current study. In general, most differentially expressed genes had low fold-changes following *C. jejuni* inoculation within each line. This is probably due to the fact that *C. jejuni* is a commensal bacterium in the chicken and is not invasive in chicken gut.

### 1. Genetic difference *between* lines

Global gene expression profile. Genetic and environmental components contribute to disease resistance in chickens with differences in susceptibility found in a number of diseases including avian leukosis, infectious bronchitis, infectious bursal disease, Marek's disease, salmonellosis and coccidiosis [24]. The two broiler lines used in this study have been evaluated in *Salmonella*, *Enterococcus* and *C. jejuni* challenge studies [7], [25], [26], [27] and in all instances chickens from line A were more resistant than line B chickens. Collectively, these studies show that these two genetic lines maintain a similar resistance pattern in response to different pathogens although they were not selected for resistance to any specific pathogen.

Although more differentially expressed genes were found between line A and line B, fewer GO BP terms were significantly enriched for those differentially expressed genes. Most of these differentially expressed genes result from genetic differences between these two lines. In addition, the genes with higher expression in line B from enriched GO BP were mainly associated with metabolic processes including the insulin receptor signaling pathway. The results from the GO BP suggest that upon inoculation with *C. jejuni*, line B chickens respond with a dramatic up-regulation in lipid, glucose, and amino acid metabolism undoubtedly for use in the response to the colonization. Chickens devote considerable resources and machinery towards self-maintenance including a network of leukocytes specialized to identify and mitigate challenges to self-maintenance. The consequences of ineffective self-maintenance include diminished productivity and dominance by pathogens [28], [29], [30]. The costs of development of the host defenses come primarily from the expenditure of energy to fuel the inefficient process of an effective immune response and to provide substrates (e.g., amino acids and lipids) for the production of effector leukocytes that protect the bird from infections [31]. The fact that line B chickens must initiate a rapid metabolic response to counter an colonization confirms our earlier results demonstrating the inefficiency of the innate immune cell functional activities in these birds probably due to directing resources to growth [25], [26], [32], [33], [34], [35].

Immune-related gene profile. Although a majority of the differentially expressed genes between the two lines were related to metabolic function, several immune-related genes, such as SOCS2, SOCS3, IL 7, NALP1, and β-defensins 10 and 12 were also differentially expressed (Table 1). Intestinal epithelial cells represent the first line of defense against pathogenic bacteria in the lumen of the intestine. Besides acting as a physical barrier, epithelial cells orchestrate the immune response through the production of several innate immune mediator molecules including β-defensins. Defensins are small peptides composed of cysteine-rich cationic molecules with broad-spectrum antimicrobial activity against bacteria, fungi and certain enveloped viruses [36]. Fourteen β-defensins have been described in chickens [37], [38]. β-defensin 3, 4, 8, 13, and 14 gene expression were shown to be significantly down-regulated during *C. jejuni* inoculation in chicken cells *in vitro* [39]; whereas β-defensin 2 and 3 are up-regulated during *C. jejuni* infection in human intestinal epithelial cells [40]. In contrast to the present findings, β-defensin 10 and 12 gene expression were not affected by *C. jejuni* inoculation in chicken peripheral blood leukocyte [39]. Here, both β-defensins 10 and 12 gene expression were significantly up-regulated in the ceca of AI birds when compared to the ceca of BI birds. These results denote the importance of the role β-defensins as part of the local intestinal host response in the resistance of line A birds to *C. jejuni* colonization when compared to line B birds.

Another interesting finding in the present studies was the up-regulation of the NALP1 gene in the ceca from the line A birds. The NALP1 inflammasome, which was the first nucleotide binding and oligomerization domain (NOD)-like receptor (NLR) family molecular platform to be identified, is relatively widely expressed and is composed of NALP1, an adaptor known as apoptosis-associated speck-like protein containing card (ASC), and caspase-1 [41], [42].

These proteins are thought to function as sensors that detect conserved microbial components in intracellular compartments, similar to the role of Toll-like receptors (TLRs) at the cell surface and within endosomes. Activation of inflammasomes occurs by recognition of ligands through leucine-rich repeats (LRRs) present in the NALP proteins. This finding of the up-regulation of the NALP1 gene is noteworthy for two reasons: (1) this is the first time that a NLR receptor has been reported to be involved in host defense against *Campylobacter* and (2) the up-regulated expression of the NALP1 gene in the ceca of line A birds when compared to line B birds demonstrates the importance of the recognition systems is in the resistance/susceptibility of chickens to *Campylobacter*. Furthermore, the related cytokines interleukin (IL)-1β and IL-18 are generated as cytosolic precursors that require cleavage by the cysteine protease caspase-1 to generate biologically active IL-1β and IL-18. In the present study, we found only a down-regulation of the IL-1β gene expressed in the ceca from line A birds when compared to line B birds and no change in gene expression in IL-18 in either line. Further studies will be required to evaluate the role of these cytokines in the host response to *Campylobacter*. However, it is possible that by the time of tissue collection post-inoculation in these studies (day 7), any changes in cytokine gene expression may have been missed in the array and qRT-PCR analysis. Future studies will include a kinetic evaluation of IL-1β and IL-18 gene expression.

### 2. Host response to *C. jejuni* colonization *within* lines

#### Global gene expression profile

One of the major objectives in this study was to compare the gene expression changes within each line of chickens in the response to *C. jejuni* inoculation. In the current study, a significant response was found between *C. jejuni*-inoculated chickens and non-inoculated chickens in the gene expression profiles within the ceca of each chicken line. Following *C. jejuni* inoculation, there were more differentially expressed genes within line B than within line A, specifically more up-regulated genes (962 vs. 392, Figure 2).

Likewise, there was a major difference in the enriched GO terms within the two lines. Within line A, lymphocyte activation and lymphoid organ development were specifically enriched. Immunoregulatory networks play a pivotal role in modulating immune responses to pathogens in the intestine. To preserve tissue integrity, complementary strategies are in place, including specialized lymphocytes and antigen-presenting cell populations. Regulatory T cells (Tregs) are a central component of this regulatory network by controlling both innate and adaptive immune responses [43]. Tregs integrate with other cellular and molecular components to control immune responses and are critical for intestinal immune homeostasis [44], [45], [46].

A certain degree of constitutive effector response and inflammation is beneficial for the host, not only to maintain integrity of the tissue but also to allow the host to develop protective responses when required. This implies that the steady state regulation of this environment relies on the maintenance of a balance of antagonistic signals allowing the induction and maintenance of various classes of effector lymphocytes. Indeed, at steady state, the gut is home to a large number of lymphocytes that have the capacity to produce regulatory (IL-10 or TGF-b) cytokines. Therefore, we hypothesize that the activation of Treg population of the ceca is responsible for the enriched functional GO activity of lymphocyte activation and lymphoid organ development that was observed. These results indicate a yet another different localized response to *C. jejuni* colonization in chickens in line A that may play a role in increased resistance to bacterial colonization.

Interestingly, circadian rhythm functional term was significantly enriched with extremely high fold enrichment (98.4) in line A. Circadian rhythms are daily oscillations of multiple biological processes driven by endogenous clocks. Circadian rhythms are known to influence the immune response of mammals through their effects on the circulation of the blood as related to diurnal sleeping/waking and activity cycles. In fact, many immune parameters, such as the number of different subtypes of circulating immune cells and the level of cytokine production in response to infection with bacteria and viruses, have been well documented to display a circadian pattern in mammals [47], [48]. In humans, blood cell compartmentalization, such as with peripheral cell counts of neutrophils, T-lymphocyte subsets, B lymphocytes, monocytes, and natural killer (NK) cells, displays a circadian fluctuation across the day [49]. The peak of each subtype of cells in peripheral blood varies with time. The numbers of monocytes, B cells, and T cells reach maximum value during the sleep phase, whereas neutrophils, NK cells, and activated T cells peak during the waking phase. Generally, these phenomena have been attributed to neuroendocrine circuits involving hormonal mediators, such as cortisol, melatonin, and insulin-like growth factor. A similar oscillation has also been observed in rodents [47], [50]. Thus, the circadian immunological parameters which affect activity in both humans and rodents are well conserved under baseline physiological conditions, indicating parallel clock control mechanisms for the human and mouse immune systems.

In mammals, the molecular apparatus governing circadian rhythms has been elucidated to comprise a transcription-translation feedback loop involving more than 12 genes, including *Period2* (*Per2*) [51], [52]. The *Period 2* (*Per2*) gene is a key molecular component in controlling mammalian circadian rhythms at the levels of gene expression, physiology, and pathogenesis. However, the basic features of molecular clock components in the immune system and the role of clock genes in regulating host immune defenses remain uncharacterized.

Daily rhythmicities are well known in the chicken, and include rhythms in daily egg laying, calling at dawn, and daily changes in physiological functions such as metabolic rate [53], brain temperature [54], heart rate [55] and ovulation [56]. In poultry, circadian rhythms are generated by a transcription-translation-based oscillatory loop that involve clock genes, including *Per2* (Period 2) and *Per3*, *Clock*, and *Bmal1* (brain and muscle Arnt-like protein 1) [57], [58], [59]. PER2 forms part of a complex of proteins that inhibits the transcriptional activator that promotes the transcription of clock-controlled genes. Clock genes in quail and chickens have high homologies with those in mammals [56].

The data from the present study is the first report providing evidence for a role of circadian rhythms in poultry resistance to *C. jejuni* colonization. In addition, validation of two genes from this functional term by qRT-PCR further confirmed the potential role of circadian clock in the host response to C. *jejuni* inoculation. Most importantly, this is further evidence of a unique mechanism of host defenses in line A birds. Understanding the nature of the circadian clock in the immune system, its role in immune regulation, and avian host defenses is critical for the advancement of our knowledge of immune function which can be used to benefit therapeutic efforts. Because cytokines that are produced by lymphocytes and macrophages are potent mediators of immune responses and the levels of individual cytokines can determine immune effector mechanisms, understanding immune-circadian clock control of specific immune mechanisms may have important applications poultry genetics and resistance to pathogens.

Based on the global gene expression analysis, within line B chickens, colonization with C. *jejuni* resulted in considerable down-regulation of genes that encode the immune response, immune system development, inflammatory response, epithelial cell regulation and epithelial cell proliferation; all of which are involved in the local response to infection. The gastrointestinal (GI) tract is the largest interface between an animal's internal milieu and its exterior environment. As such, it forms a physical barrier between both environments. However, the function of the GI tract in the well being of an animal is more complex than this passive role. The GI tract not only regulates the selective entry of nutrients while keeping vigilant against pathogens but also is largely responsible for shaping the immune response. Through specialized receptors and other more general mechanisms, the GI tract is not only able to sense changes in its environment but also to actively respond to these changes. These responses allow the intestine to contribute to the defense against microbes and to the control and regulation of the local immune response. The intestinal epithelium is a sensor of the luminal environment, not only controlling digestive, absorptive, and secretory functions, but also relaying information to the mucosal immune, vascular and nervous systems. The intestinal epithelium as a critical component of a communications network that is essential for transmitting signals generated in response to infection with microbial pathogens to cells of the innate and acquired immune systems in the underlying intestinal mucosa. The gut-associated lymphoid tissue (GALT) embraces a crucial component of the total immunological capacity of the host in recognizing and selectively handling alien antigens for the initiation of immune responses. The GALT constitutes the largest mass of immune cells in the body and provides specific host defense. Close, tightly orchestrated interactions between the intestinal epithelium and the GALT system are critical for normal intestinal absorptive and immunological functions. Nowhere is this interdependence between the innate and acquired systems more pertinent than at the mucosal surface of the GI tract which contains the largest number of immune cells and the highest concentration of pathogens and potential pathogens, but also harmless dietary antigens and large populations of commensal bacterial flora [60]. Thus, the mucosal immune system must be tightly controlled to assess and respond to antigens to which it is exposed and mount an appropriate effector or regulatory response [60], [61]. The present data imply that *C. jejuni* colonization of the B line induces a localized suppression of both the innate (epithelial cell regulation, inflammation) and the adaptive responses that allows the bacteria to colonize the ceca. We speculate that the dramatic up-regulation in lipid, glucose, and amino acid metabolism found in line B birds may well be an attempt to shift resources away from growth to local host defenses. This localized suppressive response within line B birds is remarkably different to that observed in line A birds that appears to up-regulate the local T cell response. Further experiments are required to further characterize and delineate the local responses within each of these lines of chickens.

#### Immune-related gene profile

Within both lines of chickens, most immune function genes were down-regulated genes following *C. jejuni* inoculation. The results provide no clear immunological-mediated mechanisms for the differential ability of birds *within* a line to be colonized. Further experiments are planned to look more in depth at pathogen recognition and intracellular signaling pathways that mediate the differential heterophil innate immune response that characterize these two lines of chickens.

### Conclusions

Gene expression profiling between two genetic lines and host response to *C. jejuni* inoculation were evaluated at the molecular level. This transcriptome approach allowed us to obtain a global overview of genes and the functional entities involved in the cecal response to *C. jejuni* colonization in two genetically distinct broiler lines. In summary, a dramatic differential host response was observed between these two lines of chickens. The more susceptible line B chickens responded to colonization with *C. jejuni* with a dramatic up-regulation in lipid, glucose, and amino acid metabolism undoubtedly for use in the response to the colonization with little or no change in immune host defenses. However, in more resistant line A birds the host defense responses were characterized by an up-regulation lymphocyte activation, probably by regulatory T cells and an increased expression of the NLR recognition receptor NALP1. Interestingly, circadian rhythm genes appear play a critical role in host response to *C. jejuni* colonization in the resistant A line. To our knowledge, this is the first time each of these responses has been observed in the avian response to an intestinal bacterial pathogen. The novel findings in several functional terms related to genetic differences and the local host response to *C. jejuni* colonization has provided a solid foundation to further characterize and define the cellular and molecular mechanisms of *C. jejuni* colonization in chickens.

## Materials and Methods

### Ethics Statement

These studies were approved by the Institutional Animal Care and Use Committee (IACUC) at Texas A&M University (AUP#2006-234), which meet all federal requirements, as defined in the Animal Welfare Act (AWA) and the Public Health Service Policy (PHS) and the Humane Care and Use of Laboratory Animals.

### Chickens, *C. jejuni* inoculation, and sample collection

Two broiler lines, A and B, were obtained from a commercial breeding company. The bacterial inoculation and sample collection were performed as described previously [7]. In brief, *C. jejuni* strain 5088 was enriched in Bolton broth (Oxoid, Basingstoke, UK) at 42°C for 40 h. Within each line, 80 one-day-old chickens were orally inoculated with 0.5 ml inoculants for a final dose of 1.8×10^5^ cfu per chicken, and 40 chickens from each line were mock inoculated with Phosphate Buffered Saline (PBS) as controls.

Both inoculated (I) and non-inoculated (N) birds were sacrificed at day 7 post-inoculation (pi). The cecal contents were collected and significantly higher bacterial cfu was found in line B (3.50 log10 cfu) than line A (1.39 log10 cfu) based on the number of bacteria in cecal contents [7]. Because both cecum and cecal tonsil are important lymphoid tissues, which have direct interaction with *C. jejuni* in cecum, the cecum including cecal tonsil was aseptically harvested and immediately immersed in 10 volumes of RNAlater (Ambion, Austin, TX) for isolation of total RNA.

### Total RNA isolation, experimental design, sample labeling, and hybridization

A 15–20 mg sample was removed from RNAlater-stabilized cecum tissue, cut into pieces and placed in a 2 ml centrifuge tube containing 600 µl Qiagen RNeasy Mini Kit lysis buffer (Qiagen, Valencia, CA). The PRO200 homogenizer (PRO Scientific, Oxford, CT) was used to homogenize the lysate. Total RNA was isolated from each homogenized sample and treated with TURBO DNA*free*™ Kit (Ambion, Austin, TX) according to the manufacturer's protocol. Forty individual RNA samples were isolated from each inoculated line (AI: inoculated line A, BI: inoculated line B) and twenty from each non-inoculated lines (AN: non-inoculated line A, BN: non-inoculated line B), in total, 120 individual RNA samples were isolated. Five samples in each group were randomly selected to make a pool with equal amounts of RNA. Eight pools were made in each inoculated line (AI and BI) and four in each non-inoculated line (AN and BN), in total, 24 pools were made.

Chicken 44k Agilent microarray was used in the current study. Pair-comparison was performed in the current study to provide four different comparisons: line A vs. line B (AN/BN and AI/BI) and inoculated vs. non-inoculated (AI/AN, BI/BN) and eight biological replicates were used in each comparison with dye balance except AN/BN (four biological replicates).

A 400 ng sample of total RNA from each pooled sample was used for labeling. A pool labeled with Cy3 or Cy5 was hybridized with another pool labeled with Cy5 or Cy3 and then incubated at 65°C for 17 h. The post-hybridization washes were performed according to the manufacturer's recommendation. The labeling, hybridization and washing procedures were followed according to Agilent's recommendation and described in detail previously [18].

### Microarray data analysis

Before normalization, signal intensity of each probe was filtered against negative controls in the microarray. Different comparisons were made between two lines (AN/BN and AI/BI) and between inoculated and non-inoculated within each line (AI/AN and BI/BN). Data normalization was performed using locally weighted scatter plot smoothing (LOWESS) [62], [63] by R project (http://www.r-project.org). The normalized natural log intensities were analyzed using a mixed model by SAS (SAS, Cary, NC) with fixed effect of treatment (I or N), line (A or B) and dye (Cy5 or Cy3) and random effect of slide and array. A *P*<0.01 was considered as significant. Minimum Information About a Microarray Experiment (MIAME) information about this experiment has been deposited in NCBI's Gene Expression Omnibus (GEO) [64]. The accession numbers are: platform: GPL6413; series: GSE10257.

Functional annotations for those differentially expressed up- and down-regulated genes were performed through the DAVID 2008 [19]. Statistics related to over representation of functional categories were performed using DAVID, which is based upon a Fisher Exact statistical methodology similar to that described by Al-Shahrour et al [65].

### Quantitative real-time PCR

Quantitative real-time PCR (qRT-PCR) was performed as described previously [18] with the listed primers (Table S6). Briefly, 1 µg of total RNA was reverse-transcribed into cDNA using random hexamers and Thermoscript™ RT-PCR system (Invitrogen, Carlsbad, CA). qRT-PCR reagents were loaded by Eppendorf ep*Motion* 5070 workstation (Eppendorf, Westbury, NY). The amplification was performed as 1 cycle of 95°C for 10 min, 40 cycles of 59°C for 15 s and 59°C for 1 min using SYBR Green Master Mix and ABI Prism 7900HT system (Applied Biosystems, Foster City, CA). The chicken β-actin gene was used as the internal standard to correct the input of cDNA. Triplicate qRT-PCRs were performed on each cDNA and the average Ct was used for further analysis. The relative quantification values were calculated using the 2^-ddCt^.

## Supporting Information

Table S1 List of signficantly expressed genes in the comparison of AN/BN
(0.32 MB XLS)

Table S2 List of significantly expressed genes in the comparison of AI/BI
(0.29 MB XLS)

Table S3 List of significantly expressed genes in the comparison of AI/AN
(0.14 MB XLS)

Table S4 List of significantly expressed genes in the comparison of BI/BN
(0.26 MB XLS)

Table S5 Relative expression level of genes shared between AI/AN and BI/BN
(0.04 MB XLS)

Table S6 Sequences of primers used in qRT-PCR
(0.03 MB XLS)
